# Effects of body-weight supported treadmill training and electrical stimulation on functional ambulation and strength in patients with incomplete traumatic spinal cord injury: A randomized controlled trial

**DOI:** 10.12669/pjms.42.1.12127

**Published:** 2026-01

**Authors:** Amir Zeb, Beenish Mehmood, Arif Shah, Shakil Ur Rehman, Muhammad Kashif

**Affiliations:** 1 Amir Zeb, BSPT, PP-DPT, MSPT. Director Rehabilitation/ Vice Principal , College of Physical Medicine & Rehabilitation, Paraplegic Center Peshawar, Pakistan, Riphah College of Rehabilitation Sciences, Riphah International University, Karachi, Pakistan; 2 Beenish Mehmood, DPT, MSPT. Physical Therapist, College of Physical Medicine & Rehabilitation, Paraplegic Center Peshawar, Pakistan; 3 Arif Shah, DPT, MSPT. Physical Therapist/ Adjunct Lecturer, College of Physical Medicine & Rehabilitation, Paraplegic Center Peshawar, Pakistan; 4 Prof. Shakil ur Rehman, PhD, Riphah College of Rehabilitation Sciences, Riphah International University, Karachi, Pakistan; 5 Prof. Muhammad Kashif, Riphah College of Rehabilitation Sciences, Riphah International University, Karachi, Pakistan

**Keywords:** Body weight supported treadmill training, Conventional therapy, Functional ambulation, Strength, electrical stimulation, Spinal cord injury

## Abstract

**Objectives:**

This study aims to evaluate the efficacy of body weight-supported treadmill training (BWSTT) combined with electrical stimulation (ES) in enhancing functional ambulation and lower limb strength.

**Methodology:**

This single-center, multi-arm randomized controlled trial with a parallel group design was conducted from July 2024 to February 2025 at the Paraplegic Center Peshawar (PCP). Eighty-one patients with incomplete traumatic spinal cord injury (SCI) were randomized into three groups: (I) BWSTT + ES + Conventional Therapy (CT), (II) BWSTT + CT and (III) CT only. Interventions were conducted for 12 weeks, with assessments at baseline, 6 weeks and 12 weeks using Walking Index for Spinal Cord Injury (WISCI-II), 6-Minute Walk Test, 10-Meter Walk Test, lower extremity muscle strength (LEMS) and spinal cord independence measure (SCIM). Repeated-measures ANOVA and post-hoc analyses with Bonferroni adjustment were performed.

**Results:**

All groups showed significant improvements in walking ability (WISCI-II) and functional independence (SCIM) (p < 0.001), with BWSTT + ES + CT demonstrating the greatest gains. At 12 weeks, this group had significantly higher WISCI-II scores than BWSTT + CT (p = 0.030) and CT only (p = 0.005). Strength and endurance improvements were greatest in the BWSTT + ES + CT group, but between-group differences were not statistically significant. Overall, the effects on the 6MWT, 10MWT, LEMS, and strength (measured via dynamometer) were higher in the BWSTT + ES + CT group; however, no significant differences were observed between groups.

**Conclusion:**

All three groups demonstrated improvements in ambulatory function and functional independence over time, with the BWSTT + ES + CT group showing the greatest overall gains. . Combining BWSTT with ES and CT showed superior results in walking ability and functional independence in individuals with incomplete SCI, while strength and endurance improvements were similar across different rehabilitation approaches.

**Registration No.** (Trial ID: 70844; IRCT ID: IRCT20230615058487N1).

## INTRODUCTION

In recent years, several innovative interventions have emerged to enhance ambulatory recovery, including functional electrical stimulation (FES), task-specific physiotherapy, and body weight-supported treadmill training (BWSTT), delivered either robotically or manually in individuals with incomplete spinal cord injury (iSCI).^1^,^2^ BWSTT reduces body load during treadmill walking, promoting safer gait training and outperforming overground therapy in speed, balance, and posture improvements. Long-term use may sustain physical function with low complication risk.^3^ It is also effective across various populations with muscle weakness, enhancing cardiovascular fitness and walking ability.^4^-^6^ Biofeedback-assisted lower limb training further improves gait and strength.^7^ However, some studies found no significant differences in outcomes like gait speed or FIM scores despite extended BWSTT use.^8^

FES has also demonstrated promising results in restoring both limb mobility and other functions, lost due to SCI, such as respiratory, bladder, bowel, and sexual functions through controlled electrical stimulation (ES) that activates paralyzed muscles. It has shown greater efficacy than manual assistance or orthoses.^9^ Despite promise, the evidence on FES and BWSTT alone or combined is limited and inconsistent, often due to small samples, varied protocols, and lack of standardized outcomes.^2^,^10^,^11^ Many studies neglect integrated approaches and suffer from methodological flaws.

Despite widespread recommendations for interventions such as BWSTT, robotic-assisted gait training (RAGT), and FES, their relative effectiveness remains unclear. Generally, overground gait training dominated SCI rehabilitation, but it has increasingly been replaced by BWSTT and RAGT with or without manual assistance or FES. While robotic devices help reduce therapist fatigue and enable more consistent training, the literature offers contradictory results, especially regarding the superiority of one method over another. Literature reveals that studies have often grouped together patients with varying levels of injury or excluded key support modalities such as FES or manual assistance. Therefore, this study aimed to investigate the combined effects of BWSTT and FES on functional ambulation and muscle strength in individuals with incomplete traumatic SCI.

## METHODOLOGY

This single-center, multi-arm randomized controlled trial with a parallel group design was conducted from July 2024 to February 2025 at the Paraplegic Center, Peshawar (PCP). The trial was prospectively registered with the Iranian Registry of Clinical Trials on July 29, 2023 (Trial ID: 70844; IRCT ID: IRCT20230615058487N1).

### Ethical Approval:

The study received ethical approval from the Research and Ethics Committees of the Faculty of Rehabilitation and Allied Health Sciences (FRAHS), Lahore, and institutional permission from the Paraplegic Center Peshawar-PCP (DIR/CPMR-PCP/2032023; dated: March 20, 2023).

The sample size was calculated based on an effect size of d = 0.332 (strength, measured using the Manual Muscle Testing [MMT] score), as reported in a previously published study 12 with a significance level of 5% and a power of 80%. Allowing for a 10% dropout rate, the required sample size was determined to be 79. Ultimately, 81 participants were enrolled to ensure 27 individuals per group.

Spinal cord injury (iSCI) patients aged 16–55 years were enrolled. Participants were randomly assigned to three groups. Group-I received BWSTT for 60 minutes per day, three times per week, in combination with ES (ES; 30 minutes twice daily, six sessions per week) and CT (60 minutes per day). Group-II underwent BWSTT (60 minutes/day, 3 times/ week) along with CT (60 minutes/day), while Group-III received only CT (60 minutes/day).

To assess the impact of the interventions, the following outcome measures were employed: the Walking Index for Spinal Cord Injury II (WISCI-II) to evaluate ambulatory function; the 6-Minute Walk Test (6MWT) for walking ability, aerobic capacity, and endurance; and the 10-Meter Walk Test (10MWT) for walking speed. Additional assessments included the Lower Extremity Motor Score (LEMS) using a dynamometer, the American Spinal Injury Association (ASIA) scale, and the Spinal Cord Independence Measure (SCIM) to evaluate ambulation and activities of daily living (ADLs). Muscle groups exhibiting weakness (MMT score ≤3), which could not be reliably assessed using a dynamometer, were evaluated using the MMT scale. These measures were recorded at baseline, mid-intervention, and post-intervention. Participants were allocated into three groups using a computer-generated simple randomization procedure in R software, where all 81 participant IDs were randomly shuffled and then assigned sequentially into three equal groups. The study followed a single-blind design: both participants and the data analyst were blinded to group allocation to minimize bias. Participants were kept blind by withholding information about group allocation and providing all interventions in a uniform clinical setting to prevent recognition of treatment differences.

### Statistical Analysis:

The study used SPSS version 22 and GraphPad Prism version 8 for data analysis. Continuous variables were summarized as mean ± SD for normally distributed data or median with interquartile range for non-normally distributed data. Normality was assessed via Shapiro-Wilk test. Categorical variables were reported as frequencies and percentages. Associations between categorical variables were evaluated using Chi-square or Fisher’s exact test. Comparisons between groups were done using one-way ANOVA or Kruskal–Wallis H test. Within-group comparisons were conducted using Repeated-measures ANOVA. Post hoc analysis was performed with Bonferroni adjustment. A p-value < 0.05 was considered statistically significant for all analyses.

## RESULTS

The mean age of the patients was 34.93 ± 11.96 years. The majority were male (82.70%). The most common causes of injury were fall from height (32.10%), firearm injury (24.69%) and road traffic accidents (23.46%). The average duration since injury was 35.02 ± 38.80 months. Regarding the type of paralysis, 87.65% had paraplegia. In terms of neurological level, in the cervical spine most patients had a level of “C5 ASIA D (54.55%)” with “T12 ASIA C (21.43%)” in the thoracic spine and “L1 ASIA C (21.43%)” in the lumbar spine. The maximum patients represented with lumbar spine injury (n=56) followed by thoracic (n=14) and cervical spine (n=11).

The demographic and clinical characteristics were comparable across the three patient groups (BWSTT+ES+CT, BWSTT+CT and CT), with no statistically significant differences observed in gender, age, cause of injury, or type of paralysis. Similarly, there were no statistically significant differences among the three groups across functional outcomes, SCIM scores, walking tests (6MWT, 10MWT), or LEMS (Table-I).

**Table-I:** Sociodemographic, clinical characteristics, and descriptive information of the study sample: incomplete traumatic spinal cord injured patients

Characteristics		Group of patients			p-value
		BWSTT+ES+CT	BWSTT+CT	CT	
Gender, n (%)	Male	22(81.48)	22(81.48)	23(85.19)	0.917
	Female	5(18.52)	5(18.52)	4(14.81)
Age (years) (Mean±SD)		35.15±11.21	36.44±12.25	32.96±12.52	0.523
Cause of Injury, n (%)	Coal mine accident	0	1(3.70)	0	0.068
	Fire arm injury	12(44.44)	3(11.11)	5(18.52)	
	Fell in well	2(7.41)	0	0	
	Fall from height	7(25.93)	11(40.74)	8(29.63)	
	History of falling object	1(3.70)	5(18.52)	6(22.22)	
	Road traffic accident	5(18.52)	6(22.22)	8(29.63)	
	Stab injury	0	1(3.70)	0	
Duration of injury (months) (Mean±SD)		36.40±40.16	43.16±50.28	25.49±18.07	0.666
Type of paralysis, n (%)	Tetraplegia	2(7.41)	3(11.11)	5(18.52)	0.450
	Paraplegia	25(92.59)	24(88.89)	22(81.48)
Walking Independence (Mean±SD)		8.26±4.46	9.51±5.09	8.81±5.14	0.693
Self-care (Mean±SD)		17.74±4.66	18.70±2.64	17.74±4.27	0.693
Respiration and Sphincter Management(Mean±SD)		31.41±10.47	30.96±11.99	30.59±11.79	0.966
Mobility (Mean±SD)		21.04±7.75	20.78±7.73	19.37±9.46	0.734
Total SCIM (Mean±SD)		70.19±19.56	70.44±20.33	67.70±22.68	0.978
6MWT (Mean±SD)		87.85±74.66	109.48±103.40	79.81±65.65	0.558
10MWT (Mean±SD)		0.30±0.26	0.29±0.25	0.32±0.28	0.955
LEMS–Total(Mean±SD)		22.48±10.37	24.37±13.50	24.96±15.24	0.771
LEMS –Right(Mean±SD)		11.74±6.80	11.48±7.23	11.63±7.55	0.991
LEMS –Left(Mean±SD)		10.74±7.21	12.89±7.67	13.33±8.29	0.424
Hip Flexor L2 (Right) (Mean±SD)		0.19±0.18	0.12±0.15	0.17±0.18	0.468
Hip Flexor L2 (Left) (Mean±SD)		0.16±0.18	0.17±0.18	0.17±0.17	0.960
Knee Extensor L3 (Right) (Mean±SD)		0.18±0.17	0.14±0.16	0.19±0.21	0.612
Knee Extensor L3 (Left) (Mean±SD)		0.20±0.21	0.19±0.15	0.22±0.20	0.544
Dorsiflexor L4 (Right) (Mean±SD)		0.06±0.24	0.11±.34	0.023±0.05	0.922
Dorsiflexor L4 (Left) (Mean±SD)		0.03±0.06	0.07±0.20	0.05±0.07	0.851
Big Toe Extensor L5 (Right) (Mean±SD)		0.02±0.08	0.03±0.11	0.01±0.01	0.812
Big Toe Extensor L5 (Left) (Mean±SD)		0.01±0.02	0.01±0.04	0.01±0.02	0.917
Planterflexor S1 (Right) (Mean±SD)		0.05±0.09	0.07±0.08	0.08±0.09	0.671
Planterflexor S1 (Left) (Mean±SD)		0.06±0.09	0.08±0.07	0.07±0.09	0.633
Total Dynamometer		0.97±0.79	1.00±1.02	0.98±0.87	0.850

A two-way RM-ANOVA was conducted with time (baseline, week 6, week 12) as the within-subject factor and group (Group-I, Group-II, Group-III) as the between-subjects factor. The analysis assessed the main effects of time and group, as well as the time × group interaction for each functional outcome measure. WISCI-II scores showed a significant main effect of time, F (1.72, 134) = 85.3, p < 0.001, and a significant time × group interaction, F (4, 156) = 8.48, p < 0.001, indicating that improvements over time varied across groups. Post-hoc comparisons at 12 weeks revealed that group-I showed significantly greater improvement compared to group-II (p = 0.030) and Group III (p = 0.005), while the difference between group-II and group-III was not statistically significant. For the 6MWT and 10MWT, a significant main effect of time was observed, F (1.91, 149) = 35.4, p < 0.0001 and F (1.91, 149) = 35.4, p < 0.001 respectively, indicating improvement across all groups. However, neither the main effect of group nor the interaction effect reached significance (p > 0.05). For the SCIM subcategory of respiration and sphincter management, there were significant main effects of time, F (1.35, 105) = 53.9, p < 0.001, and group, F (2, 78) = 5.85, p = 0.004, along with a significant time × group interaction, F (4, 156) = 21.5, p < 0.001. Post-hoc analysis at week 12 showed that only group-I had significantly greater improvement compared to group-III (p < 0.001). The self-care, mobility subdomain, and total SCIM score also showed a significant main effect of time (p< 0.05), with no significant main effect of group or interaction. Regarding LEMS, both right and left limb scores and total LEMS demonstrated significant improvements over time (all p < 0.0001), with no significant group effects (Table-II).

**Table-II:** Comparison of functional outcomes and LEMS scores at baseline and after 12 weeks among the three groups.

Characteristics	Group of patients												p-value
	BWSTT+ES+CT (n=27)				BWSTT+CT (n=27)				CT (n=27)			
	Baseline	12-week	Diff	d	Baseline	12-week	Diff	d	Baseline	12-week	Diff	d	
WISCI-II	8.26±4.46	15.00±3.06	-6.74	1.6	9.51±5.09	12.37±4.21	-2.85	0.99	8.81±5.14	11.81±3.98	-3.00	0.97	Time: p<0.001 Time ×group: P<0.001
6MWT	87.85±74.66	156.76±89.89	-68.91	1.13	109.48±103.40	171.93±105.79	-62.45	1.50	79.81±65.65	130.70±91.75	-50.89	0.95	Time: P<0.001 Time ×group: P=0.456
10MWT	0.30±0.26	0.49±0.36	-0.19	1.34	0.29±0.25	0.48±0.36	-0.19	0.75	0.32±0.28	0.50±0.40	-0.18	0.58	Time: P <0.001 Time ×group: P=0.951
Self-care-SCIM	17.74±4.66	19.81±0.62	-2.07	0.47	18.70±2.64	19.81±1.84	-1.11	0.42	17.74±4.27	19.22±4.22	-1.48	0.29	Time: P=0.001 Time ×group: P=0.822
Mobility-SCIM	21.03±7.75	24.81±4.84	-3.78	0.49	20.78±7.73	23.56±4.55	-2.78	0.58	19.37±9.46	21.89±6.74	-2.52	0.36	Time: P<0.001 Time ×group: P=0.476
Respiration & Sphincter Management- SCIM	31.41±10.47	38.22±4.11	-6.81	0.73	30.96±11.99	38.00±4.39	-7.04	0.75	30.59±11.79	36.92±8.51	-6.33	0.49	Time: P<0.001 Time ×group: P<0.001
Total SCIM	70.18±19.56	82.85±7.35	-12.67	0.73	70.44±20.33	81.37±8.86	-10.92	0.78	67.70±22.68	78.03±17.75	-10.33	0.45	Time: P<0.001 Time ×group: P=0.911
LEMS-Right	11.74±6.80	15.85±7.08	-4.11	1.15	11.48±7.23	14.55±7.30	-3.07	1.04	11.63±7.55	14.81±7.42	-3.18	0.92	Time: P<0.001 Time ×group: P=0.614
LEMS-Left	10.74±7.21	15.26±7.44	-4.52	1.35	12.89±7.67	15.81±7.79	-2.92	0.97	13.33±8.29	16.22±7.89	-2.89	0.96	Time: P<0.001 Time ×group: P=0.141
LEMS-Total	22.48±10.37	31.11±11.78	-8.63	1.62	24.37±13.50	30.37±13.83	-6.00	1.10	24.96±15.24	31.04±14.68	-6.07	1.07	Time: P<0.001 Time ×group: P=0.203

d= Cohen’s d.

Hip flexor strength (L2) on both the right and left sides improved significantly over time across all groups, as shown by the RM-ANOVA (right side: F (1.54, 120) = 30.9, p < 0.001; left side: F (1.59, 124) = 26.0, p < 0.001), with no significant differences between groups or interactions, indicating similar patterns of improvement regardless of the intervention. Although descriptive data suggested improvements in knee extension, dorsiflexion, big toe extension, plantarflexion and total dynamometer readings across all groups, no statistically significant differences were observed. Overall, the results demonstrate a significant time effect on hip flexor strength but no evidence that the different treatment protocols produced differential benefits (Table-III).

**Table-III:** Comparison of lower limb muscle strength (measured via dynamometry) across three intervention groups.

Dynamometer	Groups of patients												p-value
	BWSTT+ES+CT (n=27)				BWSTT+CT (n=27)				CT (n=27)				
	Baseline	12-week	Diff	d	Baseline	12-week	Diff	d	Baseline	12-week	Diff	d	
Right Hip flexion L2	0.19±0.175	0.27±0.18	-0.08	0.156	0.12±0.15	0.25±0.15	-0.13	0.155	0.17±0.18	0.26±0.17	-0.08	0.119	Time: p<0.001 Time ×group: P=0.448
Right Knee extension L3	0.18±0.17	0.28±0.19	-0.10	0.184	0.14±0.16	0.26±0.19	-0.11	0.184	0.19±0.21	0.28±0.21	-0.08	0.122	Time: P<0.001 Time ×group: P=0.826
Right Dorsiflexion L4	0.06±0.24	0.13±0.32	-0.06	0.223	0.11±.34	0.14±0.37	-0.02	0.068	0.023±0.05	0.08±0.25	-0.06	0.244	Time: P=0.022 Time ×group: P=0.704
Right Big toe extension L5	0.02±0.08	0.03±0.10	-0.01	0.092	0.03±0.11	0.02±0.10	0.00	0.037	0.01±0.01	0.01±0.01	-0.00	0.014	Time: P=0.567 Time ×group: P=0.448
Right Planter flexion S1	0.05±0.09	0.11±0.10	-0.05	0.074	0.07±0.08	0.10±0.09	-0.03	0.056	0.08±0.09	0.12±0.10	-0.04	0.071	Time: P<0.001 Time ×group: P=0.535
Left Hip flexion L2	0.16±0.18	0.29±0.18	-0.13	0.162	0.17±0.18	0.28±0.18	-0.11	0.169	0.17±0.17	0.24±0.19	-0.08	0.119	Time: P<0.0001 Time ×group: P=0.5655
Left Knee extension L3	0.20±0.21	0.28±0.19	-0.08	0.178	0.19±0.15	0.26±0.19	-0.07	0.167	0.22±0.20	0.28±0.21	-0.05	0.094	Time: P<0.001 Time ×group: P=0.677
Left Dorsiflexion L4	0.03±0.06	0.11±0.36	-0.08	0.330	0.07±0.20	0.15±0.36	-0.07	0.297	0.05±0.07	0.06±0.08	-0.02	0.042	Time: P=0.049 Time ×group: P=0.608
Left Big toe extension L5	0.01±0.02	0.01±0.02	-0.00	0.022	0.01±0.04	0.03±0.10	-0.02	0.097	0.01±0.02	0.01±0.02	-0.00	0.014	Time: P=0.177 Time ×group: P=0.540
Left Planter flexion S1	0.06±0.09	0.12±0.09	-0.05	0.083	0.08±0.07	0.12±0.08	-0.04	0.055	0.07±0.09	0.12±0.09	-0.05	0.076	Time: P<0.001 Time ×group: P=0.586
Total Dynamometer	0.97±0.79	1.66±1.18	-0.69	0.838	1.00±1.02	1.63±1.29	-0.63	0.702	0.98±0.87	1.48±0.97	-0.49	0.441	Time: P<0.001 Time ×group: P=0.571

d= Cohen’s d

## DISCUSSION

A review concluded that BWSTT offered no clinically meaningful improvements in walking speed compared to overground gait training, indicating it offered no added benefit over conventional therapy or no intervention.^13^ Our results align, showing no significant speed improvement in either the BWSTT + ES group (0.30 ± 0.26 to 0.49 ± 0.36) or the BWSTT-only group (0.29 ± 0.25 to 0.48 ± 0.36). Similarly, Arroyo-Fernández et al. found BWSTT no better than traditional physiotherapy.^14^ However, our study showed that combining BWSTT with ES enhanced walking speed, endurance, and daily functioning more effectively than BWSTT or conventional therapy alone. Mehrholz et al. suggested BWSTT might increase walking distance, but their findings were inconclusive due to wide confidence intervals.^13^,^15^ In contrast, our BWSTT + ES group showed greater walking performance improvements than other groups.

A review of seven studies using the 6MWT found no significant benefit of BWSGT over control interventions.^11^,^16^-^21^ The non-significant pooled effect was driven by heterogeneity in population characteristics, inconsistent intervention duration and intensity, variability in control treatments, and differences in BWS modalities across studies. In contrast, BWSTT + ES in our study led to greater gains (87.85 ± 74.66 to 156.76 ± 89.89) compared to BWSTT + CT (109.48 ± 103.40 to 171.93 ± 105.79) and conventional therapy (79.81 ± 65.65 to 130.70 ± 91.75).

Dobkin and Mehrholz reported minimal improvements in walking ability with BWSTT,^3^,^15^ consistent with our BWSTT-only group (9.51 ± 5.09 to 12.37 ± 4.21). In contrast, BWSTT + ES led to greater gains (8.26 ± 4.46 to 15.00 ± 3.06).

Some studies showed non-significant FIM outcomes or lacked statistical analysis.^16^,^18^ One trial comparing BWSTT + FES to other modalities found no increase in adverse events, indicating safety and feasibility, though our results showed greater efficacy with BWSTT + ES. The Field-Forte study found ES-assisted overground (OG) training more effective than treadmill approaches.^22^ However, our findings favored BWSTT + ES, with higher speed effect sizes (1.34 vs. 0.75) and greater improvements in walking speed, suggesting different modality responses. Musselman et al. reported better outcomes with overground walking than treadmill training,^23^ while our data showed BWSTT + ES yielded higher gains in independence and endurance.

Field and Postans found BWSTT + FES did not significantly improve speed, with a pooled difference of -0.03 m/s, consistent with our BWSTT + ES effect size of 0.19. BWSTT alone also showed minimal improvement across studies,^12^,^16^,^22^,^24^ with a pooled mean difference of 0.03 m/s. Field et al. also found no improvement in walking capacity with BWSTT 22, which contrasts with our BWSTT + ES group’s gains (87.85 ± 74.66 to 156.76 ± 89.89). Similarly, studies comparing BWSTT to other intervention strategies also demonstrated no improvement in walking capacity.^16^,^22^,^24^ FES-assisted ambulation improves endurance, reduces body-weight support needs, and enhances mobility in incomplete SCI, as reflected in our BWSTT + ES group’s increased walking distance and WISCI-II scores (8.26 ± 4.46 to 15.00 ± 3.06).^25^

Hitzig et al. reported SCIM mobility gains with FES-assisted BWSTT over 12 months.^26^ Hesse et al. showed improved speed and endurance using BWSTT + FES for quadriceps and hamstrings,^27^ supporting our results. Other studies also confirm lasting gait improvements post-FES.^28^ While Mehrholz’s review found no significant improvement in walking independence with BWSTT or BWSTT + FES,^15^ our study showed significant WISCI-II gains with BWSTT + ES (8.26 ± 4.46 to 15.00 ± 3.06; E.S = 1.6). Dobkin reported similar null results.^16^ This inconsistency may be attributed to differences in intervention intensity, participant characteristics (e.g., injury severity), outcome sensitivity (WISCI-II use), study quality, and ES protocol optimization.

In another study with a different population, virtual reality treadmill training (VRTT) surpassed standard treadmill methods, mirroring our findings where BWSTT + ES yielded higher muscle strength gains (E.S = 0.838) than BWSTT alone (0.702) or conventional therapy (0.441).^29^ In chronic hemiparesis, BWSTT + FES improved strength, balance, and gait, consistent with our results (muscle strength: 0.97 ± 0.79 to 1.66 ± 1.18; WISCI-II: 8.26 ± 4.46 to 15.00 ± 3.06).^30^

These findings indicate that combining BWSTT, ES, and CT leads to superior improvements in walking ability and functional independence in people with iSCI. While strength and endurance gains were observed across all groups, the addition of ES primarily enhanced ambulatory outcomes rather than general muscle strength. This supports the integration of multimodal, task-specific rehabilitation to maximize functional recovery.

### Limitations

The study has limitations, including an unequal gender distribution due to limited female patient availability, a lack of equal representation of paraplegic and tetraplegic patients, and the absence of advanced rehabilitation interventions like Lokomat-assisted training, aquatic therapy, and visual imagery in Pakistan. Comparative studies are needed to determine the most effective treatment strategies in low-resource settings, considering regional differences in culture, mindset, and physical characteristics.

## CONCLUSION

Combining BWSTT with ES and CT significantly improves walking independence, endurance, and lower limb muscle strength in individuals with iSCI, compared to BWSTT or CT alone. While earlier studies offered mixed results regarding the efficacy of such interventions, our findings support the added value of ES in enhancing functional outcomes. Future research with a double-blind design should focus on larger, more inclusive trials comparing BWSTT with ES to other innovative modalities, while also assessing long-term benefits and implementation feasibility in resource-limited settings.

### Author’s contributions:

**AZ, BM, & AS:** Conception, ideas, design, processing & data collection and acquisition.

**BM & AS:** Data analysis and interpretation/results.

**AZ, BM & AS:** Manuscript drafting and writing**.**

**SR & MK:** Supervision, addition of important intellectual content

**AZ, BM, AS, SR & MK:** Language editing, critical revision.

All authors have read and approved the paper. The principal investigator is responsible and accountable for the accuracy or integrity of the work.
